# Psychological aspects of pain prevention

**DOI:** 10.1097/PR9.0000000000000926

**Published:** 2021-05-05

**Authors:** Emma Fisher, Christopher Eccleston

**Affiliations:** a Department for Health, Centre for Pain Research, University of Bath, Bath, United Kingdom; b Cochrane Pain, Palliative and Supportive Care Review Group, Oxford, United Kingdom; c Department of Experimental-Clinical and Health Psychology, Ghent University, Ghent, Belgium

**Keywords:** Pain, Prevention, Cognitive behavioural therapy, Evidence, Psychological

## Abstract

How to prevent the onset, maintenance, or exacerbation of pain is a major focus of clinical pain science. Pain prevention can be distinctly organised into primary, secondary, and tertiary prevention. Primary prevention describes avoiding hurt or pain, secondary prevention describes reducing pain when pain is unavoidable, and tertiary prevention describes preventing or reducing ongoing negative consequences such as high functional disability or distress due to chronic pain. Each poses separate challenges where unique psychological factors will play a role. In this short review article, we highlight psychological factors important to primary, secondary, and tertiary prevention and provide direction for the field. We present 2 case studies on secondary prevention in children and adolescents and tertiary prevention in adults with chronic pain. Finally, we provide research directions for progression in this field, highlighting the importance of clear theoretical direction, the identification of risk factors for those most likely to develop pain, and the importance of treatment.

## Abstract 2

Psychological factors play an important role in the prevention of primary, secondary, and tertiary pain across the lifespan.

## 1. Introduction

Humans have evolved to avoid hurt because it is highly correlated with harm.^6,7,39^ Cognition and behaviour are well organized and used across multiple contexts to avoid both immediate and future painful injury. The psychology of pain prevention can usefully be described as primary, secondary, or tertiary. In pain, primary prevention is the avoidance or escape from a stimulus highly likely to cause hurt, normally through harm. Secondary prevention focusses on mitigating or reducing unavoidable harm. Tertiary prevention focusses on reducing the adverse consequences of unavoidable or unalterable pain.^19^ This distinction is important in the psychology of prevention because the goals of prevention strategies can sometimes conflict. For example, if needle procedure pain is successfully avoided, then the risk of later painful diseases is inadvertently increased. Alternatively, if the discomfort of exercise during rehabilitation from low back pain is avoided, then there is an increased risk of reduced quality of life because of unalterable disability. Success at the behavioural prevention of both hurt and harm is under the control of multiple psychological factors including, but not limited to, exposure to threat, learning, affect, cognitive ability, and social context.

In this short article, we offer an introduction to a selection of psychological factors we consider important from a clinical translation point of view. We do not attempt an exhaustive or empirical review but instead offer a narrative of which factors can be considered malleable and so open to intervention from all health care professionals who interact with those in pain or at risk of being in pain. First, we present a framework for psychological intervention for the prevention of pain. Second, we focus in detail on 2 specific examples of psychological prevention of pain: (1) the secondary prevention of chronic pain by postinjury intervention and (2) the tertiary prevention of long-term disability and distress by intervention with a chronic pain population. Third, we present research directions for further study.

## 2. A framework for psychological pain prevention

**Figure 1.**
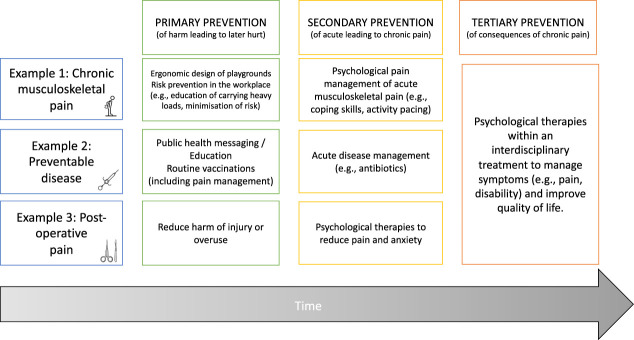
Examples for prevention of primary, secondary, and tertiary pain with an emphasis on psychological strategies.

Any comprehensive framework for psychological pain prevention has to span both the general and specific influences on behaviour and capture the multiple targets of behaviour change. Figure 1 offers a schematic of this framework showing the multiple points of possible intervention for chronic musculoskeletal pain, preventable disease, and chronic postsurgical pain.

First, there is a large literature on general health promotion and behaviour change with a goal of reducing the onset of painful disease, and here the general models and theories of behaviour change are relevant insofar as they focus on improving living standards, promoting education, and reducing exposure to tobacco, alcohol, and sedentary behaviour.^16,27,28^ This focus on general risks is rarely discussed in pain management, although general health behaviour is an important determinant of health outcomes that directly affect those in pain. Optimising general health is a useful strategy for the primary prevention of painful injury and the secondary prevention of the painful consequences of injury and disease. Health psychology offers plentiful guidance on the promotion of what are sometimes called lifestyle behaviours, although they are not always under the control of personal choice.^17^

Second, where known risks of painful injury occur, then the primary prevention of injury becomes the goal. Here there is a large and relevant literature on the psychology of risk taking in which we attempt to understand the context and decision making around risk of injury.^17^ Those interested in the ethnography or medical anthropology of self-injurious behaviour can learn from the rich qualitative evidence given at orthopaedic clinics around the world. People often willingly expose themselves to harm in the pursuit of a contextually strong higher goal, such as “social belonging” or “thrill-seeking.”^35^ Models that consider these contextual factors around uncharacteristic health risks as well as habitually safe behaviours are likely to be the most useful. Not all risk behaviour is psychologically driven: injury prevention has historically focused on workplace settings in which the demands of particular roles create risks to be mitigated by organizational intervention.

Third, turning to pain management risks, there are specific environments in which pain is deliberately induced, chosen, or deemed to be a necessary and unavoidable part of the activity. It has been hypothesized that pain experienced during such repeatedly undertaken activities is reinterpreted.^11^ Nonclinical examples are of extreme sport in which people seek the thrill of pain in pursuit of a personal achievement^6^ in body decoration, such as tattooing,^22^ or for cosmetic enhancement. However, there are many interesting clinical examples in which pain is considered unavoidable. These range from the routine and scheduled, such as dental hygiene procedures and needle procedures, to the common occurrence of postsurgical pain. What these clinical and nonclinical examples have in common is the shift in goal from an avoidance of pain to a management of pain as tolerable and a mitigation of risk of harm. This balance of exposure to pain and harms is particularly challenging when pain assessment is complex and difficult due to a lack of ability to report symptoms.^10^

Fourth, staying with injury-related pain, preonset there is often a clear opportunity to intervene. As with pre-emptive analgesia, psychological preparation for interventions that result in pain can focus either on improving compliance with a painful procedure or improving postinjurious outcome, or rather outcomes. For example, in the postoperative settings, outcomes CAN INCLUDE reduction in patient pain or anxiety, reduction in postoperative medication use, or reduction in time to discharge. Recently, there have emerged common psychological features implicated across conditions and interventions that seem to play a role in postoperative outcomes. General anxiety and pain-specific anxiety are both associated with a fear of (re)injury and pain.^13,24,43^ In addition, more recently, heightened, intrusive, repetitive worry about pain—cast as catastrophizing about pain—has emerged as a strong predictor of postsurgical outcomes^45^ and hence a target of intervention.

Fifth, when primary and secondary prevention were missing or have failed, tertiary prevention of long-term negative consequences of pain becomes the goal. More than any other this is a well-populated field in pain psychology. The goals of psychological pain management are largely unrelated to symptom control. Reducing pain is not typically a primary goal of standard cognitive behavioural therapy, with its focus on the reduction of distress and disability, specifically reducing interference and promoting social role performance.^40^ We use a language of goal pursuit and valued behaviour: a common feature at this stage is to promote an acceptance of the presence of pain in life and shift in focus to reducing the detrimental consequences of multiple complex losses.^42^ Casting psychological pain management in a prevention framework is useful inasmuch as it draws attention to the need to mitigate or prevent further decline.

Sixth, prevention in a palliative care setting focusses on complex management of total pain^33^ and a wider focus on systemic distress, often going beyond the patient to the family and medical team. The prevention or mitigation of the negative influence of self-blame and guilt, fear, and grief is an interesting area of study and place where the psychology of prevention could play a role.

By way of explication we offer 2 brief case studies to consider how a psychology of pain prevention can alter patient outcomes: one paediatric on the secondary prevention of pain after injury and one on the tertiary prevention of the negative consequences of living with chronic pain.

## 3. Case study: secondary prevention of pain after injury

Injuries are common in the community; adults as well as children trip, fall, break bones, and report muscle sprains. Before the age of 18 years, 30% of children will have sought medical attention for a fractured bone.^3^ There are more traumatic injuries that are less frequent, such as those occurring in motor vehicle accidents, where immediate and often lifesaving treatment is needed. Most people who have acute injuries will recover within a normal healing period, usually less than 3 months. However, 35% of children and adolescents go on to report chronic pain.^25^ Chronic pain in this instance is defined as pain persisting at 3 months after injury or surgery, beyond the usual time of healing.^41^

Understanding which patients are most likely to develop chronic pain after acute injury to prevent the onset of chronic pain is a critical research question in the field, and one that was most recently highlighted in a *Lancet Child & Adolescents Health Commission* on paediatric pain.^10^ Longitudinal studies to identify risk factors at the time of injury are underway in child and adolescent musculoskeletal injuries, and some work has been undertaken with children undergoing surgery. In one of the first prospective longitudinal studies investigating risk factors of persistent pain in children and adolescents after a musculoskeletal injury, Holley et al.^25^ found that patients with poorer pain conditioning and females were more likely to develop persistent musculoskeletal pain. In addition, higher depressive symptoms at the first time point were predictive of pain-related disability and poorer health-related quality of life at 3 months.^25^

Another body of literature that has investigated secondary pain prevention is the postsurgical literature. Prevalence estimates of postsurgical chronic pain coalesce around 20% in children and adolescents^32^ but estimates in adults range from 5% to 85%.^21^ This is defined as pain lasting longer than 3 months that is not associated with other surgical factors.^34^ A systematic review of prospective risk factors of developing chronic postsurgical pain in children found that presurgical pain intensity, child anxiety, child pain efficacy, and parental pain catastrophizing were predictive of chronic postsurgical pain.^32^ These factors map closely to what has been found in adults.^20^

Psychosocial variables constitute modifiable targets, either directly or indirectly, which might alter patient outcomes. For example, in paediatrics using tools borrowed from tertiary prevention,^36^ attempts are starting to be made at screening patients for high risk factors of poor pain outcomes providing the possibility of selecting them for preparatory intervention, but results are not available yet. Perioperatively, there could also be focus on altering expectations of pain after intervention or its threat value. Postoperatively, education and guided reassurance can be given on pain. Furthermore, the whole care pathway could be examined from the initial assessment and decision to refer the child for a painful intervention right up to follow-up.

Often, research has focused solely on the child, their symptoms, and personal risk factors. However, with the growing recognition of social factors, considering parental factors is essential. For example, research has shown that parental protective behaviours are associated with higher pain intensity and disability in children with chronic pain and are mostly perceived as maladaptive in the context of chronic pain.^5,18^ However, protective behaviours are generally perceived as adaptive for primary and secondary prevention of pain. The transition between acute and chronic pain is one based on duration of pain, but it is unlikely that parents behaviour will shift with this transition. Therefore, we should be sensitive about how we assess and interpret behaviours and cognitions that are adaptive in one context but considered maladaptive in another. Further measurement development is needed in this area. Moving beyond the role of parents, there is little work conducted on the role of peers after injury and social support. For most minor injuries, children will be encouraged to return to school. Social support from friends immediately after and throughout recovery may affect long-term outcomes, in that more or higher-quality social support is likely to be associated with better long-term outcomes.

Technologies of paediatric pain prevention focussed on risk analysis and mitigation, on perioperative practice, or on early augmented postoperative rehabilitation will need to be examined in randomised controlled trials, with supporting observational and economics analyses to ensure that any effective outcomes are pragmatic and able to be adopted.

## 4. Case study: the tertiary prevention of long-term disability and distress associated with chronic pain

Chronic pain is a common and disabling condition affecting 20% of adults, children, and adolescents.^4,23^ Waiting lists to attend tertiary care are often long, with patients having to wait up to 6 months and beyond for consultation and treatment.^26^ During this time, spontaneous recovery is extremely rare and symptoms such as pain severity, functional disability, and quality of life do not improve.^26^ The primary goal of pain management in tertiary care is to prevent future suffering by altering behaviour believed to be maladaptive and helping patients to pursue meaningful (valued) goals and pursuits despite pain. In essence, what is commonly believed of as an immediate treatment to reduce current suffering is focussed on the mitigation of future disability, distress, and social isolation.

There is a range of therapies that have been subject to randomised controlled trials with patients with chronic pain, but the most commonly delivered, with the largest evidence base, is cognitive behavioural therapy. Other types of therapies for which there is some evidence, albeit fewer studies in which one could assess the efficacy and harm, include behavioural therapy, acceptance commitment therapy, psychodynamic therapy, and emotional disclosure therapy. For the latter 3, there is only very low-quality evidence available. Nevertheless, all therapies delivered to patients with chronic pain ultimately have the goal to prevent the long-term negative consequences of pain.

The latest systematic review assessing the evidence base of psychological therapies for adults with chronic pain shows that when compared with active control, cognitive behavioural therapy reduces pain intensity, disability, and distress posttreatment but benefits are not maintained at follow-up.^44^ These outcomes are rated as moderate quality of evidence, meaning that further research is likely to have an important impact on our confidence in the estimate of effect and may change the estimate. One outcome, disability at follow-up, was assessed as low-quality meaning further research is very likely to have an important impact on our confidence in the estimate of effect and is likely to change that estimate. When compared with treatment as usual, benefits are found for all 3 outcomes both at posttreatment and follow-up, with quality ranging from low to moderate. Adverse events are important to consider but are poorly reported within psychological trials, and there have been growing calls for better reporting.^31^

For children and adolescents, similar findings emerge. Psychological therapies reduce pain intensity and disability posttreatment, and reductions in disability are maintained at follow-up.^14,15^ No changes are seen in anxiety or depression, but nor do most treatments deliver content to change these outcomes and these outcomes are not reported in every trial. Similar to the evidence in adults, adverse events are poorly reported and so it is difficult to truly understand any harms in trials, although from the evidence that is reported, there are few.^14,15^

Despite the relatively strong evidence for psychological therapies, or at least cognitive behavioural therapies, improvements can still be made to the treatments delivered to patients who have chronic pain. Personalised medicine is likely to define medical advances in the 21st century as we move towards tailoring our interventions more specifically to the patient in need. Better evidence for psychological interventions, beyond cognitive behavioural therapy would be a useful step here. Furthermore, as already mentioned, the current therapies being delivered do not attempt to change emotional functioning and very few address or assess sleep^12^, often comorbid in patients with chronic pain.^38^ We know that patients are complex, presenting with rich histories and often with clinical comorbidities. Ensuring that we have an evidence base that shows the most efficacious treatments for addressing these, in which order, will help to guide future practice across the globe. Despite needing more evidence here, there have been calls to stop producing evidence in other areas.^29^ Needless replications comparing cognitive behavioural therapy to treatment as usual should now be considered as research waste, not furthering our scientific intellect nor the field of prevention.

A significant gap in this field, however, from the perspective of prevention, is the need for longer-term outcome data. It is rare for a randomised controlled trial of a pharmacological intervention to report data past 6 weeks, so it is not surprising, perhaps, that RCTs of psychological treatments tend to have relatively short reporting time frames, with most follow-ups stopping at 12 months. There are a few examples of longer follow-up durations in the literature, but they are few and far between.^1,2^ Given the stated aims of therapy to promote long-term self-management, this gap in the evidence is regrettable.

COVID-19 has had a profound effect on health care globally and access to treatments in tertiary settings, particularly as many of the chronic pain community are vulnerable to contracting the condition.^8^ The detrimental and long-term effects of contracting COVID-19 are beginning to emerge^30^ and are likely to be categorised as a separate condition. Overnight, pain clinics have had to revise the way they work and have moved many face-to-face clinics online. This shift in health care was likely to occur, and pain clinicians have responded to the challenge. There is an increasing evidence base for therapies delivered remotely. Remote therapies can provide an alternative mode of delivery and can be upscaled quickly to deliver to many patients, providing a credible alternative to in-person therapy. The evidence base for remote therapies is growing and seems to be following a similar pattern to face-to-face interventions effective for reducing pain intensity and functional disability.^9,14^ Remotely delivered interventions can be a powerful way of reaching people unable to attend centralized pain clinics, and we must embrace these new technologies to deliver coordinated care to those in need and to prevent ongoing disability, poor quality of life, and distress.

## 5. Research directions for further study

Prevention is one of the key challenges currently facing our field and one that needs a coordinated approach. We should learn from previous advances in the field; some of which have been achieved relatively quickly with coordinated and multidisciplinary approaches, whereas others have taken longer as researchers and clinicians, siloed in specific research areas, struggle to make advances alone.

There are 3 key stages in advancing this field: (1) theory development, (2) risk factor identification, and (3) treatment delivery.

Current theoretical developments are scattered across the field with no general consensus, and no one model has been used extensively as has happened with, eg, the fear avoidance model in chronic pain.^43^ With growing research in this area, such as large cohort studies,^37^ there is a need for strong theoretical leadership to provide a basis for future research to test important hypotheses and provide some cohesion in this field.

Identifying those most at risk of developing long-term pain remains a public health challenge. Regarding primary and secondary prevention, some work has already been conducted in adults and is starting in children.^25^ The postsurgical literature has also indicated a few key targets, but more work is needed to determine the malleable and nonmalleable targets for intervention.

Treatment delivery will be the third major challenge faced by researchers and needs to be addressed across all treatment modalities and sectors. There are significant opportunities for psychological interventions, with a focus on primary and secondary injury prevention, and on identifying how current behaviour influences the later incidence of pain. Psychological treatments are likely to play an important role, as has already been shown in tertiary prevention, but they are also likely to play an important role for secondary prevention in people experiencing minor injuries. Psychological interventions are also important in primary prevention in presurgical contexts. These interventions can reduce anxiety around the surgical procedure and improve postsurgical outcomes. However, if malleable risk factors have been identified, it is possible that patients can be triaged depending on risk and complexity and appropriate treatments offered. This can range from education preprocedure or postprocedure to intensive psychological intervention delivery, working within a multidisciplinary team. Researchers and health care professionals must show that preventing chronic pain is a worthwhile service to invest in, which will bring about its own challenges within health care organisations where funding for services is competitive.

## 6. Conclusion

Prevention of pain is a major and present challenge in the field of pain that needs to be addressed across the lifespan. In a recent *Lancet Child and Adolescent Health* Commission on transformative action for paediatric pain, 4 key challenges were highlighted that are important when developing a roadmap for further study: to make pain matter to all, visible, understood, and better.^10^ For pain to be prevented at any stage, it must be recognised by health care professionals as something important enough to prevent. To do this, clinicians must understand the potential long-term consequences of any pain. Once understood to be important, health care professionals must have the knowledge and tools available to assess the pain. Here, we have briefly presented psychological factors that contribute to primary, secondary, and tertiary pain prevention. We have also provided brief research directions for future research in this area. Ultimately, we need coordinated and multidisciplinary collaboration to make major advances quickly in this field. We need to establish risk factors and develop treatments to offset pain, the development of chronic pain, or associated negative consequences of experiencing chronic pain.

## Disclosures

The authors have no conflicts of interest to declare.
